# Cardiovascular abnormalities in dogs with acute pancreatitis

**DOI:** 10.1111/jvim.16597

**Published:** 2022-11-25

**Authors:** Harry Cridge, Daniel K. Langlois, Jörg M. Steiner, Robert A. Sanders

**Affiliations:** ^1^ Department of Small Animal Clinical Sciences, College of Veterinary Medicine Michigan State University East Lansing Michigan USA; ^2^ Gastrointestinal Laboratory, College of Veterinary Medicine and Biomedical Sciences Texas A&M University College Station Texas USA

**Keywords:** arrythmia, echocardiogram, NT‐proBNP, Spec cPL, troponin

## Abstract

**Background:**

The prevalence and clinical importance of cardiac abnormalities in dogs with acute pancreatitis (AP) is unknown.

**Animals:**

Twelve dogs with AP and 60 archived serum samples from dogs with suspected AP.

**Methods:**

Two‐phase study. Phase I: Analysis of archived serum samples from dogs with clinical signs of AP and high Spec cPL concentrations. High sensitivity troponin I (TnIH) and N‐terminal pro‐B‐type natriuretic peptide (NT‐proBNP) concentrations were measured in achieved serum samples. Phase II: Prospective observational study. Dogs with AP underwent echocardiography and Holter monitoring. Serum cardiac troponin I (cTnI) and plasma NT‐proBNP concentrations were measured. Previously described disease severity indices were calculated for each dog.

**Results:**

Phase I: 41 of 60 dogs suspected of having AP had abnormally high TnIH concentrations and 13 of 60 had abnormally high serum NT‐proBNP concentrations. Higher TnIH concentrations were observed in dogs with Spec cPL concentration >2000 μg/L as compared to those with concentrations of 1000‐2000 μg/L. Phase II: 11 of 12 dogs diagnosed with pancreatitis had abnormal cTnI concentrations (median: 0.384 ng/mL, range: 0.041‐2.966 ng/mL, RI: ≤0.06 ng/mL) and 7 of 12 dogs had plasma NT‐proBNP concentrations above the reference interval (median: 971 pmol/L, range: 250‐2215 pmol/L, RI: ≤900 pmol/L). Supraventricular and ventricular ectopic beats occurred in 3 dogs. Echocardiographic abnormalities were detected in 5 dogs. Cardiovascular variables were not associated with indices of disease severity.

**Conclusions and Clinical Importance:**

Myocardial injury is common in dogs with AP, but clinical consequences appeared to be uncommon in our small cohort. Cardiac biomarkers should be interpreted with caution in dogs with AP.

**Abbreviations**
Ao: aortic
AP: acute pancreatitis
APPLE_full_
: acute patient physiologic and laboratory evaluation score
bpm: beats per minute
CAPS: canine acute pancreatitis severity score
CRP: C‐reactive protein
cTnI: ADVIA Centaur Troponin I Ultra‐assay
DCM: dilated cardiomyopathy
ECG: electrocardiogram
EF: ejection fraction
FS: fractional shortening
IVS: Iiterventricular septal thickness
LA: left atrial
LV: left ventricle
LVA: left ventricular area
LVEDV: left ventricular end‐diastolic volume
LVESV: left ventricular end‐systolic volume
LVID: left ventricular internal diameter
LVPW: left ventricular posterior wall thickness
M‐CAI: modified canine activity index
NT‐proBNP: N‐terminal pro‐B‐type natriuretic peptide
RI: reference Interval
SMOD: Simpson's method of discs
Spec cPL: specific canine pancreatic lipase immunoreactivity
SV: stroke volume
TnIH: Siemens Atellica IM high sensitivity troponin I assay

## 1 INTRODUCTION

Pancreatitis is the most common disorder of the exocrine pancreas in dogs.^1^ Pancreatic inflammation and subsequent cytokine release often result in systemic complications, which are suspected to contribute to the high case fatality rate observed in severe cases.^2^ Systemic complications of acute pancreatitis (AP) in dogs include coagulopathies, kidney injury, and lung injury.^3^, ^4^, ^5^ Cardiac manifestations of AP are well‐established in human patients and might offer prognostic information.^6^ Potential cardiac manifestations of AP in humans include alterations in cardiac output, stroke volume, conduction, and diastolic and systolic function.^7^, ^8^ Systemic biomarkers of injury are abnormal in humans with AP.^9^, ^10^ Furthermore, a major classification system for AP in humans utilizes the presence and persistence of organ dysfunction, including that of the cardiovascular system, to determine the severity of AP, thus highlighting the importance of these complications.^11^

In contrast to humans with AP, available data regarding cardiovascular abnormalities in dogs with AP is limited and consists of retrospective studies, case reports, and anecdotal information. Over 70% of dogs with high DGGR lipase activities in 1 study had abnormally high cardiac troponin I concentrations, but the reason why the clinician requested concurrent cardiac biomarker assessment in each case was not reported.^12^ In another study, cardiac troponin I concentrations were above the reference range in a small group of dogs with AP that were included as part of a noncardiac disease control group, but clinical information regarding the diagnosis of AP was not reported.^13^ Conflicting information exists regarding ECG abnormalities and their importance in dogs with AP.^14^, ^15^ The prevalence of structural abnormalities, as assessed by echocardiogram, is also unreported. The aim of our study was to further evaluate the frequency and clinical importance of cardiac abnormalities in dogs with naturally occurring AP.

## 2 MATERIALS AND METHODS

### 2.1 Study overview and AP case definition

Two‐phase study. In phase I of the study, the database of the Texas A&M Gastrointestinal Laboratory was searched from 1/4/22 to 2/23/22 to identify serum specimens from dogs with suspected AP. Cases were selected if the medical records revealed that clinical signs were consistent with AP and the measured cPLI concentration (as measured by Spec cPL, Idexx Laboratories, Westbrook, MA) was ≥400 μg/L. A total of 60 samples were selected, with 20 samples encompassing each of the following cPLI ranges: 400‐999, 1000‐2000, and >2000 μg/L. These groupings were selected to allow for evaluation of cardiac biomarker concentrations over a range of abnormal cPLI concentrations.

Dogs presenting to Michigan State University College of Veterinary Medicine with suspected AP were eligible for prospective enrollment in phase II of the study. The study protocol was approved by the Institutional Animal Care and Use Committee at Michigan State University, and informed consent was obtained from all owners. The diagnosis of AP was based on an integration of clinical signs, serum cPLI concentrations, and abdominal ultrasound findings.^16^ Dogs were required to have ≥2 of the following clinical signs: hyporexia/dysrexia, vomiting, diarrhea, lethargy, and abdominal discomfort. In addition, dogs were required to have an abnormal test result on a patient‐side pancreatic lipase assay (SNAP cPL, IDEXX Laboratories Inc, Westbrook, Maine) and ≥2 sonographic features of AP, which included pancreatic enlargement, a hypoechoic pancreatic parenchyma, and abnormal peripancreatic mesenteric echogenicity.^16^, ^17^ Dogs also had serum cPLI concentrations with an analytically valid immunoassay (Spec cPL, Idexx Laboratories, Westbrook, Maine).^18^, ^19^, ^20^, ^21^ Although quantitative cPLI results were not immediately available, any dog that was subsequently found to have a serum cPLI concentration <400 μg/dL was subsequently excluded from analysis. Other inclusion criteria included a CBC, serum chemistry profile, clotting times (prothrombin time and activated partial thromboplastin time), and venous blood gas analysis within 2‐days of enrollment to evaluate for concurrent disease. Dogs with known preexisting cardiac disease, defined as prior documentation of a murmur, abnormal cardiac findings on radiographs or echocardiogram, or electrocardiographic (ECG) abnormalities were also excluded. Dogs with a PCV ≤15% were excluded because of the sample volume required for measurement of biomarkers. Blood was obtained by jugular venipuncture, placed into standard collection tubes, and plasma and serum were harvested by routine methods.

### 2.2 Biomarkers

In phase I of the study, all dogs had serum concentrations of high sensitivity troponin‐I (TnIH) (Siemens Atellica IM High Sensitivity Troponin I Assay, Siemens Healthineers, Malvern, PA) and serum N‐terminal pro‐B‐type natriuretic peptide (NT‐proBNP; Canine Cardiopet proBNP test kit, IDEXX Laboratories Inc., Westbrook, Maine) measured via commercial assays. Serum and plasma NT‐proBNP quantification have been analytically validated for canine samples.^22^ All samples were analyzed within 1 week of being received and were handled in accordance with handling and storage recommendations by the manufacturer (−80°F).

In phase II of the study, all dogs meeting enrollment criteria had serum samples submitted for measurement of C‐reactive protein (CRP) and cardiac troponin I (cTnI) concentration and a plasma sample submitted for the measurement of NT‐proBNP concentration. The assays used for measurements of CRP (Gentian canine‐specific immunoturbidimetric CRP assay, Gentian Diagnostics ASA, Norway), cTnI (ADVIA Centaur TnI Ultra‐assay, Siemens Medical Solutions Diagnostics, New York), and NT‐proBNP (Canine Cardiopet proBNP test kit, IDEXX Laboratories Inc., Westbrook, Maine) are validated for use in dogs.^22^, ^23^, ^24^, ^25^ The TnIH assay (TnIH assay, Texas A&M Gastrointestinal Laboratory, College Station, Texas) utilized in phase I was used because the cTnI assay used in phase II was discontinued and replaced by the TnIH assay by the manufacturer. Reportedly, it has an increased sensitivity for the measurement of TnIH in humans.^26^ The TnIH assay has been validated by the laboratory, but no published validation study exists at this time. The laboratory provided cut‐off values were determined based on 39 healthy dogs with a normal echocardiogram.

### 2.3 Holter monitoring

In phase II, dogs underwent continuous Holter monitoring with a 3‐lead 3‐channel system for 48 hours (Lifecard CF Holter Monitor, Spacelabs Health Care Inc, Snoqualmie, Washington). Holter's were removed early only if the animal died or if specifically requested by the owner. The Holter tracings were analyzed by an automated system (Pathfinder Digital, Spacelabs Health Care Inc, Snoqualmie, Washington) and reports were verified by a board‐certified veterinary cardiologist (RAS). When greater than 24 hours of data was available, each 24‐hour period was analyzed separately and was only included into the analysis if there was >21 hours of usable data. Data obtained included: normal heart rate parameters (min/mean/max heart rate and number of pauses greater than 2 seconds), frequency of isolated ventricular premature complexes, ventricular couplets, ventricular triplets, and episodes of ventricular tachycardia. The frequencies of ventricular bigeminy and ventricular trigeminy were noted. The definitions of conduction disturbances used by the analysis software are included in Table 1. Supraventricular burden was calculated as the percentage of abnormal supraventricular beats during the monitoring period. Ventricular burden was calculated as the percentage of abnormal ventricular beats during the monitoring period. Continuous Holter monitoring was not performed in phase I of the study, as phase I involved only used archived serum samples.

**TABLE 1:** Definitions utilized by the analysis software

Definition	Setting
Minimum heart rate	1‐min average of the lowest sinus heart rate over a 24‐h period
Mean heart rate	1‐min average of the sinus heart rate over a 24‐h period
Maximum heart rate	1‐min average of the highest sinus heart rate over a 24‐h period
Sinus pause	Interval of ≥2 s
Dropped beat	≥200% of the prevailing NN interval
Ventricular tachycardia	≥4 consecutive ventricular premature ectopics at ≥51 bpm
Idioventricular rhythm	≥4 beats at ≤50 bpm
Ventricular couplet	2 consecutive ventricular premature ectopic beats
Ventricular triplet	3 consecutive ventricular premature ectopic beats
Ventricular trigeminy	A pattern of a ventricular premature beat as every 3rd complex ≥3 cycles
Ventricular bigeminy	A pattern with a ventricular premature beat as every 2nd complex ≥3 cycles
Supraventricular ectopic beat	<30% of prevailing NN interval when NN ≥40 bpm
Premature ventricular ectopic	<100% of prevailing RR interval
Ventricular ectopic	>100% of prevailing RR interval but <150% of RR interval
Ventricular escape beat	≥150% of the prevailing RR interval
R on T phenomenon	<170 ms + 5% prevailing NN interval

*Note*: Definitions utilized by analysis software to detect conduction disturbances. All data were subsequently reviewed and verified by a board‐certified veterinary cardiologist.

### 2.4 Echocardiography

In phase II, dogs underwent an echocardiogram (Vivid E90, GE Healthcare, Waukesha, Wisconsin) including standard 2D M‐mode, color Doppler, and spectral doppler imaging performed by 1 of 2 board certified veterinary cardiologists. All stored images and cine loops were subsequently reviewed by 1 board certified veterinary cardiologist (RAS). All echocardiograms were performed in right and left lateral recumbency in a quiet, dark room on an echocardiogram table. No sedation was utilized. Measurements included 2D aortic diameter (Ao) and left atrial diameter (LA) from the right parasternal short axis image of the heart base, as described and left ventricular area (LVA) in diastole and systole from the right parasternal short axis view.^27^ At the level of the papillary muscles from the right parasternal short axis image, interventricular septal thickness (IVS), fractional shortening (FS), left ventricular internal diameter (LVID) in systole and diastole, and left ventricular posterior wall thickness (LVPW) in systole and diastole were obtained using M‐mode measurements. Simpson's method of discs (SMOD) derived left ventricular end‐diastolic volume (LVEDV), left ventricular end‐systolic volume (LVESV), ejection fraction (EF), and stroke volume (SV) were calculated as previously described using the right parasternal long axis view. Pulsed wave Doppler was used to evaluate the blood flow velocities in the pulmonary artery from the right parasternal short axis at the level of the heart base and continuous wave Doppler was used to evaluate the aortic blood flow velocities from the left apical views. All cardiac valves were evaluated using color flow Doppler for the presence of insufficiencies. For all echocardiographic measurements 3 consecutive cycles were measured and averaged. Echocardiograms were not performed in phase I of the study.

### 2.5 Disease severity indices

Multiple severity indices have been proposed for use in dogs with AP and no studies have directly compared the utility of multiple severity indices in an independent cohort. In our study, disease severity was determined by the following indices: canine acute pancreatitis severity score (CAPS), modified canine activity index (M‐CAI), and the acute patient physiologic and laboratory evaluation score (APPLE_full_), as utilized in previous studies.^28^, ^29^, ^30^ Another severity index, called the clinical severity index, was not utilized because the assessment of cardiovascular abnormalities is a component of its total score.^14^ Severity indices were not able to be calculated for phase I of the study.

### 2.6 Statistical analysis

Data sets were assessed for normality using Shapiro‐Wilk testing and box‐plot inspection. Data were reported as medians and ranges, and nonparametric statistical testing was used because of the data distributions and sample size. For phase I of the study, Kruskal‐Wallis with Dunn's multiple comparison testing was used to compare TnIH and NT‐proBNP concentrations among dogs with various serum cPLI concentrations (ie, 400‐999, 1000‐2000 , and > 2000 μg/L). For phase II of the study, Spearman's rank correlation coefficients were calculated to evaluate potential associations of cardiac variables (eg, biomarkers, supraventricular burdens, ventricular burdens) with disease severity indices. In addition, cardiac variables were compared between those dogs with a CAPS ≥11 and those dogs with a CAPS <11. The cut‐point of ≥11 was selected because this value has been suggested to be 89% sensitive and 90% specific for predicting short‐term case fatality in dogs with AP.^28^ Statistical analyses were conducted using commercially available software (GraphPad Prism Version 9.0; GraphPad Software Inc, San Diego, California), and for all analyses, values of *P* ≤ .05 were considered significant.

## 3 RESULTS

### 3.1 Animals

In phase I of the study, the median age of dogs enrolled was 11 years (range: 1‐19 years). Thirty‐three dogs were spayed females, 22 were neutered males, and 4 were intact males. The sex of 1 dog was not recorded. The median weight of the enrolled dogs was 13.3 kg (range: 3.2‐34.0 kg). Twenty‐three dogs were mixed breed dogs, 4 were Yorkshire terriers, 3 were Boxers, and 3 were Dachshunds. Two each of the following breeds were represented: Cavalier King Charles spaniel, Cocker spaniel, Labrador retriever, Maltese, Soft‐coated Wheaten terrier, and Havanese terrier. One each of the following breeds were also represented Beagle, Boston terrier, Bulldog, Cardigan Welsh corgi, Catahoula leopard dog, Golden retriever, Jack Russel terrier, Miniature pinscher, Miniature poodle, Pointer, Shiba Inu, Shih tzu, and Standard poodle. The breed for 2 dogs were unavailable.

In phase II of the study, 19 dogs met initial enrollment criteria, but 7 dogs were subsequently removed because serum cPLI concentrations were either normal or in the diagnostic gray‐zone (200‐399 μg/dL). The median age of the 12 dogs undergoing analysis was 9.5 years (range: 8‐15 years). Seven were spayed females and 5 were neutered males. The median weight of enrolled dogs was 15.6 kg (range: 5.5‐51.0 kg). Six were mixed breed dogs and 1 each of the following breeds were represented: Brittany spaniel, Fox terrier, Golden Retriever, Labrador retriever, Shih tzu, and Yorkshire terrier. Eleven of 12 dogs survived. The median serum cPLI concentration was 1316 μg/dL (range: 491‐>2000 μg/dL).

### 3.2 Cardiac biomarkers

**FIGURE 1:**
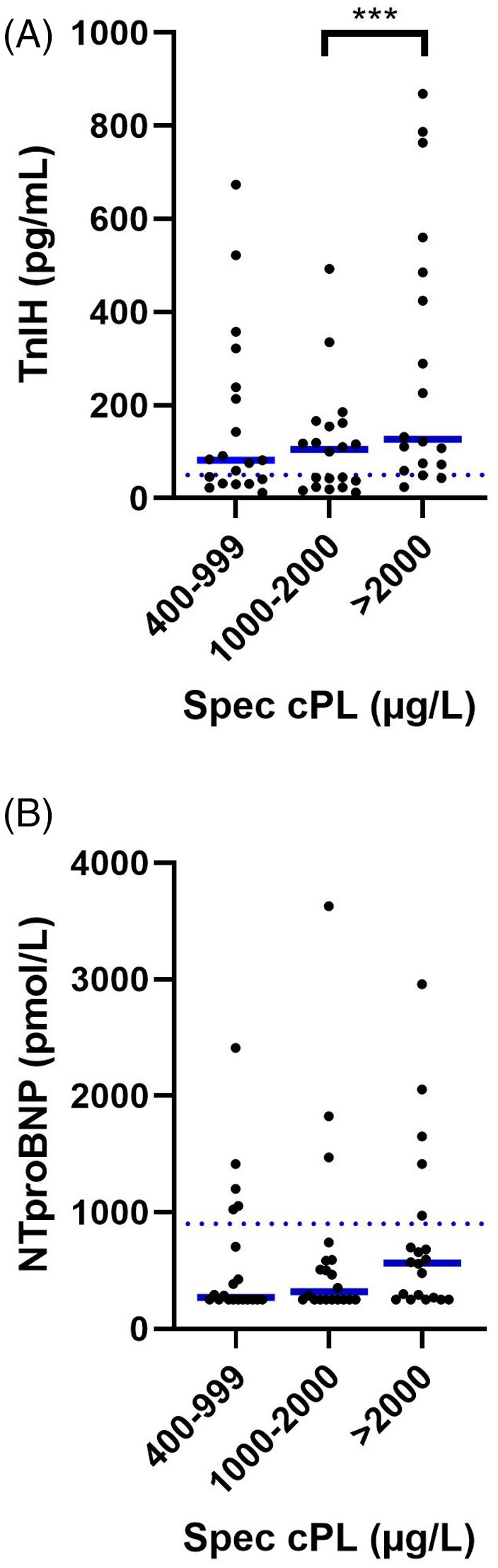
Cardiac biomarker concentrations in dogs with suspected AP (Phase I). Serum TnIH (A) and NT‐proBNP (B) concentrations of dogs enrolled in phase I of the study. Image A has 3 data points with a TnIH concentration >1000 pg/mL excluded to maintain graphical clarity. No data points have been excluded from image B. The solid blue line for each group represents the median value, and the dotted blue line across all groups represents the laboratories cut‐off value for the upper‐end of the RI. ***Statistically significant comparison

In phase I of the study the median serum TnIH was 111 ng/L (range: 12‐6096 ng/L, laboratory cut off: <50 pg/mL). Forty‐one of 60 dogs had increased serum TnIH concentrations. Twenty‐four of 60 dogs had serum TnIH concentrations above the laboratory provided cut‐off for myocardial injury (>135 pg/mL). A diagnostic gray zone has been recommended for concentrations between 50 and 135 pg/mL. The median serum NT‐proBNP concentration was 326 pmol/L (range: 250‐3629 pmol/L, RI: ≤900 pmol/L). Thirteen of the 60 dogs had increased serum NT‐proBNP concentrations. Serum TnIH and serum NT‐proBNP concentrations in relation to cPLI concentrations are shown in Table 2. Higher serum TnIH concentrations were seen in dogs with serum cPLI concentrations >2000 μg/L vs 1000‐2000 μg/L (Figure 1). No other comparisons, including those for NT‐proBNP concentrations were statistically significant.

**TABLE 2:** Cardiac biomarker concentrations in archived serum samples from phase I of the study

Spec cPL concentration	Median serum TnIH concentration (range)	Median serum NT‐proBNP concentration (range)
400‐999 μg/L	83.1 (11.7‐5874.0) pg/mL	268.0 (250.0‐2413.0) pmol/L
1000‐2000 μg/L	105.3 (12.8‐492.8) pg/mL	318.0 (250.0‐3629.0) pmol/L
>2000 μg/L	179.2 (24.6‐6096.0) pg/mL	565.0 (250.0‐2959.0) pmol/L

*Note*: Archived samples were obtained from a variety of practice types.

In phase II of the study, the median serum cTnI concentration was 0.384 ng/mL (range: 0.041‐2.966 ng/mL, laboratory cut off: ≤0.06 ng/mL). Eleven of the 12 dogs (91.7%) had increased serum cTnI concentrations. The serum cTnI concentration of the dog that died was 1.027 ng/mL. The median plasma NT‐proBNP concentration was 971 pmol/L (range: 250‐2215 pmol/L). Seven of the 12 dogs (58%) had increased plasma NT‐proBNP concentrations. The plasma NT‐proBNP concentration of the dog that died was 1096 pmol/L.

### 3.3 Holter evaluation and echocardiography

All 12 dogs were evaluated using a Holter monitor. The median supraventricular burden was 0.197% (range: 0.00%‐3.59%). The median ventricular burden was 0.022% (range: 0.00%‐4.64%). The top 3 conduction abnormalities noted included: dropped beats, premature ventricular ectopic beats, and ventricular ectopic beats. Further details on dog‐specific conduction disturbances can be found in Supplementary material S1. One dog died while wearing a Holter monitor. Four episodes of ventricular tachycardia with 2 episodes of an idioventricular rhythm after a period of bradycardia occurred around the time of death. The mean heart rate was 141 beats per minute (bpm) (range: 40‐164 bpm). Supraventricular and ventricular burdens were 0.00% and 0.17%, respectively.

Routine evaluation of stored echocardiographic images was performed by RAS. Seven of 12 dogs had no major structural abnormalities. However, mild structural abnormalities were noted in some dogs, including mild degenerative valve disease (3 dogs), mild reductions in indices of systolic function (2 dogs), pulmonary arterial hypertension (1 dog), and mild degenerative valve disease with mild pericardial effusion (1 dog).

### 3.4 Associations with disease severity indices

In phase II of the study, serum cardiac troponin I concentrations (cTnI) were correlated with CAPS (*R*_s_ = 0.618, *P* = .032), but not M‐CAI (*R*_s_ = 0.138, *P* = .668), or APPLE_full_ (*R*_s_ = 0.333, *P* = .290). Serum cardiac troponin I concentrations were not different between those dogs with CAPS ≥11 and those with CAPS <11 with a *P* = .625. No dogs in our study had an APPLE_full_ score ≥40, preventing evaluation as a dichotomous variable. Plasma NT‐proBNP concentrations were not correlated with CAPS (*R*_s_ = 0.205, *P* = .523), M‐CAI (*R*_s_ = −0.261, *P* = .413), or APPLE_full_ (*R*_s_ = −0.057, *P* = 860). N‐terminal pro‐B‐type natriuretic peptide concentrations in plasma were not significantly different between those dogs with CAPS ≥11 and those with CAPS <11 with a *P* = .812. No dogs in our study had an APPLE_full_ score ≥40, preventing evaluation as a dichotomous variable.

Supraventricular burden was not correlated with CAPS (*R*_s_ = 0.239, *P* = .436), M‐CAI (*R*_s_ = 0.171, *P* = .595), or APPLE_full_ (*R*_s_ = −0.212, *P* = .508). Supraventricular burden was not different between those dogs with CAPS ≥11 and those with CAPS <11 with a *P* = .625. No dogs in our study had an APPLE_full_ score ≥40, preventing evaluation as a dichotomous variable. Ventricular burden was not correlated with CAPS (*R*_s_ = −0.212, *P* = .509), M‐CAI (*R*_s_ = 0.068, *P* = .834), or APPLE_full_ (*R*_s_ = −0.156, *P* = .628). Ventricular burden was not correlated with dichotomous CAPS values (≥11 vs <11) with a *P* = .876. No dogs in our study had an APPLE_full_ score ≥40, preventing evaluation as a dichotomous variable. No other comparisons to disease severity indices were significant.

### 3.5 Necropsy (1 dog, natural mortality)

The dog that died in phase II of the study underwent a necropsy examination. The necropsy confirmed the presence of severe regionally extensive necrotizing and suppurative pancreatitis. The left and right ventricular wall were examined, revealing no evidence of cardiomyocyte degeneration, inflammation, or fibrosis in the examined sections.

## 4 DISCUSSION

The diagnosis of suspected AP in phase I of the study was based on history, clinical signs, and an elevated serum cPLI concentration (≥400 μg/dL). Most (41/60) archived serum samples had increased (>50 pg/mL) serum TnIH concentrations and 24 of 60 dogs had serum TnIH concentrations above the laboratory provided cut‐off value for myocardial injury (>135 pg/mL). A smaller proportion of dogs (13/60) also had increased serum NT‐proBNP concentrations. The diagnosis of AP in phase II of the study was based on a complete clinical diagnosis based on a consistent history/physical examination, diagnostic imaging findings, and an elevated serum cPLI concentration (≥400 μg/dL).^16^ One dog also had histopathologic evidence of pancreatitis. Most dogs (11/12) hospitalized with clinical AP had serum cTnI concentrations above the upper limit of the RI and over half (7/12) have plasma NT‐proBNP concentrations above the upper limit of the RI. Elevations in serum cTnI and plasma NT‐proBNP were not correlated with indices of disease severity. Despite the high frequency of increased cardiac biomarkers, dogs with AP had a low frequency of echocardiographic and electrical conduction abnormalities. The mean ventricular burden in this study was 0.02%, which indicates approximately 1 abnormal ventricular beats per hour in a dog with a heart rate of 100 bpm, although marked variation is noted. Holter data is limited in noncardiac or healthy dog cohorts, 1 study revealed that even healthy mature dogs have a small number of abnormal ventricular beats.^31^

Cardiac troponins are quantitative biomarkers that are considered the gold standard for detection of myocardial injury.^32^ In phase I of the study, we utilized a new TnIH assay, whereas in phase II of the study we utilized the prior cTnI assay.^25^ The results from these assays are not equivalent. Serum troponin concentrations are low in healthy dogs.^25^, ^33^ The median serum cTnI concentration in phase II (median: 0.384 ng/mL, range: 0.041‐2.966 ng/mL) was 22.6× greater than the median cTnI concentration reported in 2 previously published healthy dog cohorts using the same assay (median: 0.017 ng/mL, range: 0.006‐0.128 ng/mL and median: 0.017 ng/mL, range: 0.006‐0.136 ng/mL).^25^, ^33^ The median serum cTnI concentration in this study was lower than that reported for 8 dogs with AP (median: 4.85 ng/mL, range: 0.062‐30.8 ng/mL) in a study evaluating cardiac troponin concentrations in dogs with a variety of cardiac and noncardiac diseases.^13^ Potential discrepancies between this study and the prior study might relate to differences in diagnostic criteria, degree of cardiac screening, and disease severity. Next, we compared the median serum cTnI concentration from dogs with AP in phase II of this study to published diagnostic cut off points for primary cardiac disorders utilizing the same assay.^33^ As the TnIH assay is new, peer‐reviewed publications, detailing proposed cut‐offs for various clinical scenarios have not yet been published and similar comparisons cannot be made. The median cTnI concentration in AP dogs (0.384 ng/mL, range: 0.041‐2.966 ng/mL) was greater than that of dogs classified as having mild‐to‐moderate cardiac disease (median: 0.113 ng/mL, range: 0.026‐0.291 ng/mL) and just below those classified as having severe cardiac disease requiring immediate intervention (median: 0.394 ng/mL, range: 0.155‐4246 ng/mL) in a recent study.^33^ Potential causes of myocardial injury in dogs with AP include hypotension, hypoxemia, microthrombi, or toxic effects of inflammatory cytokines.^34^, ^35^, ^36^, ^37^ Despite myocardial injury only 1 dog in the study cohort died, and cTnI did not appear to be correlated with AP severity. Overall, the greatest clinical significance of these findings is that caution should be utilized when interpreting cardiac troponin concentrations in dogs with concurrent AP.

N‐terminal pro‐B‐type natriuretic peptide is a biomarker of myocardial stretch that has been validated for use in dogs.^22^ The median plasma NT‐proBNP concentration in phase II of this study was 2.6‐3.0× greater the median NT‐proBNP concentration reported in a study evaluating week to week variability of plasma NT‐proBNP concentrations in healthy dogs (weekly medians: 320, 358, 377 pmol/L [range: 159‐650 pmol/L]).^38^ The median plasma NT‐proBNP concentration was also 1.8× the median NT‐proBNP concentration reported in a second study of healthy dogs (median: 543 pmol/L, range: 16‐1558 pmol/L).^39^ The median plasma NT‐proBNP concentration was 3.9× higher than the median NT‐proBNP concentration (median: 250 pmol/L, range: 250‐1214 pmol/L) reported in a recent study of healthy Saluki dogs with normal echocardiographic measurements.^40^ Next, we compared the median plasma NT‐proBNP concentration from dogs with AP in phase II of this study to proposed diagnostic cut offs for primary cardiac disorders utilizing the same assay.^24^, ^41^, ^42^ The median plasma NT‐proBNP concentration in dogs with AP in phase II of this study was greater than the plasma NT‐proBNP (445 pmol/L) diagnostic cut‐off proposed to discriminate dogs with mitral valve disease or dilated cardiomyopathy (DCM) from healthy dogs.^24^ The median plasma NT‐proBNP concentration in this study was below the proposed diagnostic cut‐off (study 1: 1158 pmol/L, study 2:2447 pmol/L) for discrimination of cardiac from respiratory disease in 2 recent studies.^41^, ^42^ Studies utilizing alternate NT‐proBNP assays have; however, reported markedly lower diagnostic cut‐offs for discriminating cardiac from respiratory disease.^43^, ^44^ The median plasma NT‐proBNP concentration in dogs was also above the proposed diagnostic cut‐off (457 pmol/L) to identify occult DCM in Doberman Pinschers.^45^ In phase I of the study a much lower frequency of increased serum NT‐proBNP concentrations was noted (median: 325.5 pmol/L, range: 250‐3629 pmol/L). Potential explanations for the discrepancy between the results include the sample type. Serum samples utilized for phase I of the study were left‐over serum samples from cPLI measurement, whereas in phase II plasma samples were used. Serum is reportedly less stable than plasma for the measurement of NT‐proBNP.^22^ The difference in concentration is however more likely explained by differences in the study cohort. In phase II of the study samples were obtained from hospitalized dogs at a referral hospital, whereas in phase I, samples originated from multiple practice types including primary care practices. It is plausible that differences in disease severity or treatments (intravenous fluid therapy vs outpatient management) existed between these 2 cohorts. Potential causes of myocardial stretch in AP include the effects of fluid resuscitation. One study of 6 healthy dogs showed that a 90 mL/kg of isotonic crystalloid fluids provided as a bolus could increase median plasma NT‐proBNP concentrations to 797 pmol/L (range: 250‐985 pmol/L) 12 hours after fluid administration.^46^

Although this study emphasizes cautious interpretation of cardiac biomarkers in dogs with concurrent AP, it should be noted that various other noncardiac disorders have been associated with elevated cardiac biomarker concentrations, including systemic inflammatory response syndrome, gastric dilatation and volvulus, and infectious diseases.^37^, ^47^, ^48^, ^49^, ^50^

In our study we utilized a Holter monitor to investigate the prevalence of conduction abnormalities in dogs with AP. This method was selected over routine brief ECG assessments (1‐5 minutes), to avoid confounding factors such as restraint, and to allow for the detection of intermittent arrythmias. Holter monitoring has been shown to be more sensitive than routine brief ECG assessments for the detection of arrythmias in other studies.^51^ A 48‐hour monitoring period showed a mild benefit over 24‐hour monitoring in a recent study, although the benefit was predominantly restricted to identification of iterative or paroxysmal supraventricular arrythmias.^52^ Holter monitoring is routinely used in the assessment of disease severity, disease progression, and evaluation of antiarrhythmic response in veterinary cardiology.^53^, ^54^, ^55^ In this study we found low rates of supraventricular (median: 0.197%, range: 0.00%‐3.59%) and ventricular burden (median: 0.022%, range: 0.00%‐4.64%) in dogs with AP. Supraventricular and ventricular burdens were not correlated with AP disease severity indices. The most common abnormalities were dropped beats, premature ventricular ectopic beats, and ventricular ectopic beats. Additionally, review of data from the dog that died while wearing the Holter monitor, indicated no contributing arrythmia, as determined by a board‐certified veterinary cardiologist (RAS). Low rates of arrythmias have also been reported in healthy dogs in prior studies.^56^, ^57^, ^58^ Similarly, a scoring system for cardiac arrythmias was not associated with outcome in a prior retrospective study of dogs with AP.^15^ Standard echocardiography appears to detect limited abnormalities in dogs with AP. However, it is possible that more advanced imaging techniques, such as speckle tracking echocardiography, could yield more significant abnormalities in future studies.

Although not an aim of the current study, CAPS was not a useful prognostic indicator in this study cohort. A CAPS score ≥11 is reported to have a sensitivity of 89% and a specificity of 90% for predicting short term mortality (within 30 days of admission) in dogs with AP.^28^ In our study, 5 dogs had a CAPS score ≥11, of which only 1/5 died. All dogs with a CAPS score <11 and 5/6 dogs with a CAPS ≥11 survived. Previously reported disease severity markers might need to be reevaluated and directly compared in large independent cohorts of dogs with AP, to determine true prognostic performance.

A limitation of the present study was the small sample size in phase II. This could lead to a type II error and might mean that uncommon abnormalities were missed. However, it is the largest prospective evaluation of cardiovascular abnormalities of AP in dogs to date and offers a comprehensive structural, conduction, and biomarker assessment of such dogs. Additionally, phase I of the study thoroughly investigated the prevalence of cardiac biomarker abnormalities in a cohort of 60 dogs. Although only dogs with no evidence of preexisting cardiovascular disease were included in the study, given the lack of longitudinal follow up, it is possible that dogs may have gone on to develop primary cardiac disease. A further limitation of our study was the inability to evaluate the effect of cardiovascular abnormalities on mortality, given the low case fatality rate in our study cohort. The low case fatality rate may be related to either dog or treatment specific factors, or both. Two cardiac troponin assays were utilized in this study, limiting direct comparison between phase I and phase II of the study. This was unfortunately out of the control of the study investigators as 1 assay was discontinued by the manufacturer. The TnIH assay was internally analytically validated, but no external peer‐reviewed and published analytical validation study currently exists. The diagnosis of pancreatitis is notoriously challenging. In phase I of the study, we utilized dogs with clinical signs and an elevated serum cPLI concentration, without imaging data. Serum cPLI concentrations ≥400 μg/dL have a high sensitivity (81.0%‐90.9%) and specificity (74.1%‐81.1%) for the diagnosis of pancreatitis.^20^ The presence of ≥2 sonographic features of pancreatitis is reported to have a moderate sensitivity (78%) and specificity (69%) for the diagnosis of pancreatitis.^17^ The lack of ultrasound examinations in phase I might have impacted diagnostic accuracy. In phase II of the study the results of an abdominal ultrasound were integrated into the overall clinical diagnosis.

In conclusion, increased serum and plasma cardiac biomarkers were common in dogs with suspected or diagnosed pancreatitis, but this did not translate into clinically important cardiac complications. Thus, cardiac biomarkers should be interpreted with caution in dogs with pancreatitis.

## CONFLICT OF INTEREST DECLARATION

Dr. Steiner serves as the director of the Gastrointestinal Laboratory at Texas A&M University, which offers measurement of serum canine pancreatic lipase immunoreactivity concentration (cPLI, by Spec cPL), and measurement of cardiac troponin I (via TnIH) on a fee‐for‐service basis. Dr. Steiner also acts as a paid consultant for IDEXX Laboratories, which is the manufacturer of the Spec cPL assay and also offers cPLI testing using the Spec cPL on a fee‐for‐service basis. No other authors have a conflict of interest.

## OFF‐LABEL ANTIMICROBIAL DECLARATION

Authors declare no off‐label use of antimicrobials.

## INSTITUTIONAL ANIMAL CARE AND USE COMMITTEE (IACUC) OR OTHER APPROVAL DECLARATION

Approval was granted by the Michigan State University IACUC.

## HUMAN ETHICS APPROVAL DECLARATION

Authors declare human ethics approval was not needed for this study.

## Supporting information


**Appendix S1**. Supporting Information

## References

[1] Xenoulis PG . Diagnosis of pancreatitis in dogs and cats. J Small Anim Pract. 2015;56:13‐26. 25586803 10.1111/jsap.12274

[2] Papa K , Mathe A , Abonyi‐Toth Z , et al. Occurrence, clinical features and outcome of canine pancreatitis (80 cases). Acta Vet Hung. 2011;59:37‐52. 21354940 10.1556/AVet.59.2011.1.4

[3] Gori E , Lippi I , Guidi G , Perondi F , Pierini A , Marchetti V . Acute pancreatitis and acute kidney injury in dogs. Vet J. 2019;245:77‐81. 30819430 10.1016/j.tvjl.2019.01.002

[4] Nielsen L , Holm J , Rozanski E , Meola D , Price LL , Laforcade A . Multicenter investigation of hemostatic dysfunction in 15 dogs with acute pancreatitis. J Vet Emerg Crit Care. 2019;29:264‐268. 10.1111/vec.12840 31034751

[5] Gori E , Pierini A , Ceccherini G , et al. Pulmonary complications in dogs with acute presentation of pancreatitis. BMC Vet Res. 2020;16(1):1‐8. 32571307 10.1186/s12917-020-02427-y PMC7310026

[6] Yegneswaran B , Kostis JB , Pitchumoni CS . Cardiovascular manifestations of acute pancreatitis. J Crit Care. 2011;26(225):e211‐e228. 10.1016/j.jcrc.2010.10.013 21185146

[7] Wilson PM , Neoptolemos JP . Acute pancreatitis as a model of sepsis. J Antimicrob Chemother. 1998;41(Suppl A):51‐63. 10.1093/jac/41.suppl_1.51 9511087

[8] Thandassery RB , Choudhary N , Bahl A , Kochhar R . Characterization of cardiac dysfunction by echocardiography in early severe acute pancreatitis. Pancreas. 2017;46:626‐630. 28398922 10.1097/MPA.0000000000000820

[9] Bugdaci MS , Oztekin E , Kara E , Koker I , Tufan A . Prognostic value of increased B type natriuretic peptide in cases with acute pancreatitis. Eur J Intern Med. 2012;23:e97‐e100. 22560401 10.1016/j.ejim.2012.02.012

[10] Barassi A , Pezzilli R , Romanelli MC , Banfi G , Merlini G , d'Eril GM . Serum markers of myocardial damage in acute pancreatitis: a prospective time course study. Pancreas. 2015;44:678‐680. 25872135 10.1097/MPA.0000000000000320

[11] Banks PA , Bollen TL , Dervenis C , et al. Classification of acute pancreatitis—2012: revision of the Atlanta classification and definitions by international consensus. Gut. 2013;62:102‐111. 23100216 10.1136/gutjnl-2012-302779

[12] O'Brien PM . Cardiac injury detected by troponin is associated with pancreatitis detected by DGGR‐lipase in dogs and cats. Research Communications of the 25th ECVIM‐CA Congress. J Vet Intern Med. 2016;30:417.

[13] Serra M , Papakonstantinou S , Adamcova M , O'Brien PJ . Veterinary and toxicological applications for the detection of cardiac injury using cardiac troponin. Vet J. 2010;185:50‐57. 20621713 10.1016/j.tvjl.2010.04.013

[14] Mansfield CS , James FE , Robertson ID . Development of a clinical severity index for dogs with acute pancreatitis. J Am Vet Med Assoc. 2008;233:936‐944. 18795856 10.2460/javma.233.6.936

[15] Kuzi S , Mazor R , Segev G , et al. Prognostic markers and assessment of a previously published clinical severity index in 109 hospitalised dogs with acute presentation of pancreatitis. Vet Rec. 2020;187:e13. 31662578 10.1136/vr.105364

[16] Cridge H , Twedt DC , Marolf AJ , Sharkey LC , Steiner JM . Advances in the diagnosis of acute pancreatitis in dogs. J Vet Intern Med. 2021;35:2572‐2587. 34751442 10.1111/jvim.16292 PMC8692219

[17] Cridge H , Sullivant AM , Wills RW , Lee AM . Association between abdominal ultrasound findings, the specific canine pancreatic lipase assay, clinical severity indices, and clinical diagnosis in dogs with pancreatitis. J Vet Intern Med. 2020;34:636‐643. 31951054 10.1111/jvim.15693 PMC7096629

[18] McCord K , Morley PS , Armstrong J , et al. A multi‐institutional study evaluating the diagnostic utility of the spec cPL™ and SNAP® cPL™ in clinical acute pancreatitis in 84 dogs. J Vet Intern Med. 2012;26:888‐896. 22676337 10.1111/j.1939-1676.2012.00951.x

[19] Haworth MD , Hosgood G , Swindells KL , Mansfield CS . Diagnostic accuracy of the SNAP and Spec canine pancreatic lipase tests for pancreatitis in dogs presenting with clinical signs of acute abdominal disease. J Vet Emerg Crit Care. 2014;24:135‐143. 10.1111/vec.12158 24739030

[20] Cridge H , MacLeod AG , Pachtinger GE , et al. Evaluation of SNAP cPL, Spec cPL, VetScan cPL Rapid Test, and Precision PSL assays for the diagnosis of clinical pancreatitis in dogs. J Vet Intern Med. 2018;32:658‐664. 29424454 10.1111/jvim.15039 PMC5866996

[21] Huth SP , Relford R , Steiner JM , Strong‐Townsend MI , Williams DA . Analytical validation of an ELISA for measurement of canine pancreas‐specific lipase. Vet Clin Pathol. 2010;39:346‐353. 20698941 10.1111/j.1939-165X.2010.00245.x

[22] Cahill RJ , Pigeon K , Strong‐Townsend MI , Drexel JP , Clark GH , Buch JS . Analytical validation of a second‐generation immunoassay for the quantification of N‐terminal pro‐B‐type natriuretic peptide in canine blood. J Vet Diagn Invest. 2015;27:61‐67. 25525139 10.1177/1040638714562826

[23] Hillstrom A , Hagman R , Tvedten H , et al. Validation of a commercially available automated canine‐specific immunoturbidimetric method for measuring canine C‐reactive protein. Vet Clin Pathol. 2014;43:235‐243. 24798319 10.1111/vcp.12150 PMC4257579

[24] Oyama MA , Fox PR , Rush JE , Rozanski EA , Lesser M . Clinical utility of serum N‐terminal pro‐B‐type natriuretic peptide concentration for identifying cardiac disease in dogs and assessing disease severity. J Am Vet Med Assoc. 2008;232:1496‐1503. 18479239 10.2460/javma.232.10.1496

[25] Winter RL , Saunders AB , Gordon SG , et al. Analytical validation and clinical evaluation of a commercially available high‐sensitivity immunoassay for the measurement of troponin I in humans for use in dogs. J Vet Cardiol. 2014;16:81‐89. 24834862 10.1016/j.jvc.2014.03.002

[26] Chapman AR , Fujisawa T , Lee KK , et al. Novel high‐sensitivity cardiac troponin I assay in patients with suspected acute coronary syndrome. Heart. 2019;105:616‐622. 30442743 10.1136/heartjnl-2018-314093 PMC6580754

[27] Rishniw M , Erb HN . Evaluation of four 2‐dimensional echocardiographic methods of assessing left atrial size in dogs. J Vet Intern Med. 2000;14:429‐435. 10935894 10.1892/0891-6640(2000)014<0429:eofemo>2.3.co;2

[28] Fabres V , Dossin O , Reif C , et al. Development and validation of a novel clinical scoring system for short‐term prediction of death in dogs with acute pancreatitis. J Vet Intern Med. 2019;33:499‐507. 30770578 10.1111/jvim.15421 PMC6430934

[29] Keany KM , Fosgate GT , Perry SM , Stroup ST , Steiner JM . Serum concentrations of canine pancreatic lipase immunoreactivity and C‐reactive protein for monitoring disease progression in dogs with acute pancreatitis. J Vet Intern Med. 2021;35:2187‐2195. 34250650 10.1111/jvim.16218 PMC8478023

[30] Hayes G , Mathews K , Doig G , et al. The acute patient physiologic and laboratory evaluation (APPLE) score: a severity of illness stratification system for hospitalized dogs. J Vet Intern Med. 2010;24:1034‐1047. 20629945 10.1111/j.1939-1676.2010.0552.x

[31] Meurs KS , Siper AW , Wright NA , Hamlin RL . Use of ambulatory electrocardiography for detection of ventricular premature complexes in healthy dogs. J Am Vet Med Assoc. 2001;218:1291‐1292. 11330615 10.2460/javma.2001.218.1291

[32] Langhorn R , Willesen JL . Cardiac troponins in dogs and cats. J Vet Intern Med. 2016;30:36‐50. 26681537 10.1111/jvim.13801 PMC4913658

[33] Polizopoulou ZS , Koutinas CK , Dasopoulou A , et al. Serial analysis of serum cardiac troponin I changes and correlation with clinical findings in 46 dogs with mitral valve disease. Vet Clin Pathol. 2014;43:218‐225. 24612252 10.1111/vcp.12124

[34] Arlati S , Brenna S , Prencipe L , et al. Myocardial necrosis in ICU patients with acute non‐cardiac disease: a prospective study. Intensive Care Med. 2000;26:31‐37. 10663277 10.1007/s001340050008

[35] Pereira KHVP , Hibaru VY , Mata FKD , et al. Use of cardiac troponin I (cTnI) levels to diagnose severe hypoxia and myocardial injury induced by perinatal asphyxia in neonatal dogs. Theriogenology. 2022;180:146‐153. 34973646 10.1016/j.theriogenology.2021.12.027

[36] Maeder M , Fehr T , Rickli H , Ammann P . Sepsis‐associated myocardial dysfunction: diagnostic and prognostic impact of cardiac troponins and natriuretic peptides. Chest. 2006;129:1349‐1366. 16685029 10.1378/chest.129.5.1349

[37] Langhorn R , Oyama MA , King LG , et al. Prognostic importance of myocardial injury in critically ill dogs with systemic inflammation. J Vet Intern Med. 2013;27:895‐903. 23678990 10.1111/jvim.12105

[38] Kellihan HB , Oyama MA , Reynolds CA , Stepien RL . Weekly variability of plasma and serum NT‐proBNP measurements in normal dogs. J Vet Cardiol. 2009;11:S93‐S97. 19395335 10.1016/j.jvc.2009.03.003

[39] Winter RL , Saunders AB , Gordon SG , Buch JS , Miller MW . Biologic variability of N‐terminal pro‐brain natriuretic peptide in healthy dogs and dogs with myxomatous mitral valve disease. J Vet Cardiol. 2017;19:124‐131. 28111138 10.1016/j.jvc.2016.11.001

[40] Brennan C , Gunasekaran T , Sanders RA . Evaluation of plasma N‐terminal pro‐B‐type natriuretic peptide levels in healthy North American Salukis with normal echocardiographic measurements. PLoS One. 2022;17:e0260079. 35100273 10.1371/journal.pone.0260079 PMC8803176

[41] Oyama MA , Rush JE , Rozanski EA , et al. Assessment of serum N‐terminal pro‐B‐type natriuretic peptide concentration for differentiation of congestive heart failure from primary respiratory tract disease as the cause of respiratory signs in dogs. J Am Vet Med Assoc. 2009;235:1319‐1325. 19951101 10.2460/javma.235.11.1319

[42] Fox PR , Oyama MA , Hezzell MJ , et al. Relationship of plasma N‐terminal pro‐brain natriuretic peptide concentrations to heart failure classification and cause of respiratory distress in dogs using a 2nd generation ELISA assay. J Vet Intern Med. 2015;29:171‐179. 25308881 10.1111/jvim.12472 PMC4858067

[43] Boswood A , Dukes‐McEwan J , Loureiro J , et al. The diagnostic accuracy of different natriuretic peptides in the investigation of canine cardiac disease. J Small Anim Pract. 2008;49:26‐32. 18005104 10.1111/j.1748-5827.2007.00510.x

[44] Fine DM , DeClue AE , Reinero CR . Evaluation of circulating amino terminal‐pro‐B‐type natriuretic peptide concentration in dogs with respiratory distress attributable to congestive heart failure or primary pulmonary disease. J Am Vet Med Assoc. 2008;232:1674‐1679. 18518809 10.2460/javma.232.11.1674

[45] Singletary GE , Morris NA , O'Sullivan ML , et al. Prospective evaluation of NT‐proBNP assay to detect occult dilated cardiomyopathy and predict survival in Doberman Pinschers. J Vet Intern Med. 2012;26:1330‐1336. 22998090 10.1111/j.1939-1676.2012.1000.x

[46] Khoo A , Waldner CL , Carr AP , Gaunt MC . Effect of three resuscitative fluid therapy strategies on NT‐proBNP concentration in healthy dogs. J Vet Emerg Crit Care. 2019;29:143‐148. 10.1111/vec.12812 30767350

[47] Aona BD , Rush JE , Rozanski EA , Cunningham SM , Sharp CR , Freeman LM . Evaluation of echocardiography and cardiac biomarker concentrations in dogs with gastric dilatation volvulus. J Vet Emerg Crit Care. 2017;27:631‐637. 10.1111/vec.12667 28960715

[48] Kocaturk M , Martinez S , Eralp O , Tvarijonaviciute A , Ceron J , Yilmaz Z . Tei index (myocardial performance index) and cardiac biomarkers in dogs with parvoviral enteritis. Res Vet Sci. 2012;92:24‐29. 21074228 10.1016/j.rvsc.2010.10.018

[49] Lobetti RG . Cardiac involvement in canine babesiosis. J S Afr Vet Assoc. 2005;76:4‐8. 15900893 10.4102/jsava.v76i1.386

[50] Silva VBC , Sousa MG , Araujo CRA , et al. Cardiac biomarkers in dogs with visceral leishmaniasis. Arch Med Vet. 2016;48:269‐275.

[51] Meurs KM , Spier AW , Wright NA , Hamlin RL . Comparison of in‐hospital versus 24‐hour ambulatory electrocardiography for detection of ventricular premature complexes in mature Boxers. J Am Vet Med Assoc. 2001;218:222‐224. 11195827 10.2460/javma.2001.218.222

[52] Mavropoulou A , Oliveira P , Willis R . Holter monitoring in dogs: 24 h vs. 48 h. Vet J. 2021;272:105628. 33941329 10.1016/j.tvjl.2021.105628

[53] Meurs KM , Spier AW , Miller MW , Lehmkuhl L , Towbin JA . Familial ventricular arrhythmias in boxers. J Vet Intern Med. 1999;13:437‐439. 10499727 10.1892/0891-6640(1999)013<0437:fvaib>2.3.co;2

[54] Calvert CA , Jacobs GJ , Smith DD , Rathbun SL , Pickus CW . Association between results of ambulatory electrocardiography and development of cardiomyopathy during long‐term follow‐up of Doberman pinschers. J Am Vet Med Assoc. 2000;216:34‐39. 10638315 10.2460/javma.2000.216.34

[55] Meurs KM , Spier AW , Wright NA , et al. Comparison of the effects of four antiarrhythmic treatments for familial ventricular arrhythmias in Boxers. J Am Vet Med Assoc. 2002;221:522‐527. 12184702 10.2460/javma.2002.221.522

[56] Ulloa HM , Houston BJ , Altrogge DM . Arrhythmia prevalence during ambulatory electrocardiographic monitoring of beagles. Am J Vet Res. 1995;56:275‐281. 7771691

[57] Rasmussen CE , Vesterholm S , Ludvigsen TP , et al. Holter monitoring in clinically healthy Cavalier King Charles Spaniels, Wire‐haired Dachshunds, and Cairn Terriers. J Vet Intern Med. 2011;25:460‐468. 21418322 10.1111/j.1939-1676.2011.0707.x

[58] Sanders RA , Kurosawa TA , Sist MD . Ambulatory electrocardiographic evaluation of the occurrence of arrhythmias in healthy Salukis. J Am Vet Med Assoc. 2018;252:966‐969. 29595391 10.2460/javma.252.8.966

