# Supplementary material for: Cardiovascular abnormalities in dogs with acute pancreatitis
Source: J Vet Intern Med. 2022 Nov 25;37(1):28–36. doi: 10.1111/jvim.16597 (PMC9889725; doi:10.1111/jvim.16597)
Supplement: Supplementary file 1 — Appendix S1. Supporting Information [file JVIM-37-28-s001.pdf]

Supplementary Material 1: Dog specific conduction disturbances

Dog	**1***	**2**	**3**	**4**	**5**	**6**	**7**	**8**	**9**	**10**	**11**	**12**
Weight of dog (kg)	9.4	9.0	5.5	30.2	8.1	36.0	21.8	51.0	6.0	39.4	29.4	8.2
Min HR (bpm)	40	43	88	42	66	57	65	63	118	49	34	41
Mean HR (bpm)	141	83	140	81	103	86	94	87	154	79	68	101
Max HR (bpm)	164	180	202	152	209	179	197	173	196	202	169	202
Sinus pause (episodes/24h)	42	63	0	56	8	47	1	8	0	58	523	105
Dropped beat (episodes/24h)	0	90	276	99	1349	1254	1305	4678	4	279	38	14
VT (episodes/24h)	5	0	0	0	0	0	0	0	0	2	0	0
IVR (episodes/24h)	2	0	0	0	0	0	0	0	0	0	0	0
Couplet (episodes/24h)	0	0	0	0	10	0	0	0	5	6	3	0
Triplet (episodes/24h)	2	0	0	0	1	0	0	0	1	2	0	0
Bigeminy (episodes/24h)	0	0	0	0	1	0	0	0	0	0	0	0
Trigeminy (episodes/24h)	0	0	0	0	5	0	0	0	0	0	0	0
Premature V ectopic beat (episodes/24h)	1	0	0	109	5	9	5	154	4397	7	5	1
V escape (episodes/24h)	0	0	0	55	886	0	0	0	29	1	0	0
SV ectopic beat (episodes/24h)	0	0	0	54	0	0	0	0	0	0	0	0
V ectopic beat (episodes/24h)	0	0	0	0	0	0	0	0	0	1	5	0
R on T (episodes/24h)	0	0	0	0	0	0	0	0	0	0	0	0
Artifact (%)	26.9	<0.1	0	4.0	0.8	0.1	0.1	0.2	1.5	0	<0.1	0

Key: * = died
